# Genetic and genomic interventions in crop biofortification: Examples in millets

**DOI:** 10.3389/fpls.2023.1123655

**Published:** 2023-03-06

**Authors:** Himabindu Kudapa, Rutwik Barmukh, Hindu Vemuri, Sunita Gorthy, Rajasekhar Pinnamaneni, Mani Vetriventhan, Rakesh K. Srivastava, Priyanka Joshi, Ephrem Habyarimana, S. K. Gupta, Mahalingam Govindaraj

**Affiliations:** ^1^ International Crops Research Institute for the Semi-Arid Tropics, Patancheru, Telangana, India; ^2^ International Maize and Wheat Improvement Center (CIMMYT), Patancheru, Telangana, India; ^3^ Koneru Lakshmaiah Education Foundation, Vaddeswaram, Guntur, Andhra Pradesh, India; ^4^ HarvestPlus Program, Alliance of Bioversity International and the International Center for Tropical Agriculture (CIAT), Cali, Colombia

**Keywords:** micronutrients, genetic resources, genomics-assisted breeding, omics, precision phenotyping, genome editing

## Abstract

Micronutrient malnutrition is a serious threat to the developing world’s human population, which largely relies on a cereal-based diet that lacks diversity and micronutrients. Besides major cereals, millets represent the key sources of energy, protein, vitamins, and minerals for people residing in the dryland tropics and drought-prone areas of South Asia and sub-Saharan Africa. Millets serve as multi-purpose crops with several salient traits including tolerance to abiotic stresses, adaptation to diverse agro-ecologies, higher productivity in nutrient-poor soils, and rich nutritional characteristics. Considering the potential of millets in empowering smallholder farmers, adapting to changing climate, and transforming agrifood systems, the year 2023 has been declared by the United Nations as the International Year of Millets. In this review, we highlight recent genetic and genomic innovations that can be explored to enhance grain micronutrient density in millets. We summarize the advances made in high-throughput phenotyping to accurately measure grain micronutrient content in cereals. We shed light on genetic diversity in millet germplasm collections existing globally that can be exploited for developing nutrient-dense and high-yielding varieties to address food and nutritional security. Furthermore, we describe the progress made in the fields of genomics, proteomics, metabolomics, and phenomics with an emphasis on enhancing the grain nutritional content for designing competitive biofortified varieties for the future. Considering the close genetic-relatedness within cereals, upcoming research should focus on identifying the genetic and genomic basis of nutritional traits in millets and introgressing them into major cereals through integrated omics approaches. Recent breakthroughs in the genome editing toolbox would be crucial for mainstreaming biofortification in millets.

## Introduction

The current world population of 7.9 billion, increasing at an alarming rate of 1.05% annually, is anticipated to reach 10 billion by 2057 (https://www.worldometers.info/world-population/#pastfuture). To fill the hungry stomachs of the burgeoning human population, approximately 8–9 billion tons of food are produced worldwide (FAO, 2021). Despite this, global hunger affected more than 828 million individuals in 2021 and was associated predominantly with increased conflict, violence, and climate variability (FAO, 2021). Access to healthy and nutritious food will be challenging in the future, with malnutrition already influencing about one out of three people globally. The minerals that are essential for human health are classified into two major categories: (i) macronutrients that include sodium (Na), potassium (K), calcium (Ca), magnesium (Mg), sulfur (S), and phosphorus (P) and (ii) micronutrients including iron (Fe), copper (Cu), zinc (Zn), manganese (Mn), fluorine (F), iodine (I), and molybdenum (Mo), among others (Bouis and Welch, 2010). Notably, the distressingly enhancing population growth has led to more than three billion people worldwide suffering from “hidden hunger” or “micronutrient deficiencies,” wherein they fail to obtain enough nutrients or micronutrients from the foods they eat to lead healthy and productive lives. This is particularly severe in the case of children, as they fail to develop to their full mental and physical potential (HarvestPlus, 2019).

The increasing prevalence of micronutrient malnutrition is likely to have major repercussions among the poor in developing countries (particularly South Asia and sub-Saharan Africa) who rely heavily on staple crops such as maize, wheat, and rice, and eat few micronutrient-rich foods such as fruits, vegetables, and animal and fish products. For instance, the population in developing nations have an average intake of Ca less than half of that by the population that resides in developed countries (Nordin, 2000). Furthermore, Zn deficiency affects 17% of the world’s population, with the highest risk occurring in South Asia and sub-Saharan Africa, while Fe deficiency affects 32.9% of people worldwide (Kassebaum et al., 2014). In addition to vitamin A, I, Zn, and Fe deficiencies that are regarded as major health concerns globally according to The World Health Report (2000), the deficiencies of other nutrients such as folate are also placing human health at risk (Mayer et al., 2008).

To improve the nutritional status of the global human population, many intervention strategies were proposed to combat the ill effects of micronutrient malnourishment such as dietary diversification, mineral supplementation, food fortification, and biofortification (Cominelli et al., 2020; Ofori et al., 2022). For instance, dietary diversification provides diverse staple food to contribute macro- and micronutrients at the recommended dietary allowance (RDA) in a sustainable way (Lowe, 2021). Mineral supplementation pertains to the intake of extra nutrients *via* capsules, tablets, or syrups to increase the nutrient levels obtained through food consumption to contribute to meeting the RDA requirements in a short term (Tam et al., 2020). Food fortification involves adding particular nutrients to the food for increasing their nutritional content in order to aid consumers achieve the RDA for such nutrients with recurrent investments (Das et al., 2019). The aforementioned interventions have been less successful because of political, socio-economic, infrastructure-related, and technical constraints that are apparent in many developing countries. As a result, biofortification is a cost-effective, sustainable, and consumer friendly solution for meeting target levels of micronutrients such as Fe and Zn in human populations with one-time investment (Govindaraj et al., 2019; Kumar et al., 2019; Kiran et al., 2022). Biofortification is a crop-breeding process of enhancing the essential nutritional value of staple food crops as they grow, as opposed to adding nutrients while processing the edible parts into food products. This process is facilitated by conventional breeding and genetic improvement approaches that are based on natural genetic variation, modern selection methods, and the detection of novel genes and gene combinations associated with grain micronutrient content. For instance, HarvestPlus aims to develop nutritious crop varieties through biofortification to provide higher amounts of Zn, Fe, and Provitamin-A to vulnerable populations (children and women) *via* the staple food that they eat (www.harvestplus.org). To date, 290 biofortified varieties of 12 distinct staple crops (rice, wheat, maize, cassava, pearl millet, bean, sweet potato, lentil, cowpea, banana/plantain, sorghum, and Irish potato) have been released in more than 60 countries across the world (HarvestPlus, 2018). One example of successful biofortification is the vitamin A-rich orange-fleshed sweet potato that is grown in several African countries.

Taking into consideration the present scenario of worldwide nutritional insecurity, in this review, we attempt to highlight the role of cereals (particularly millets) in increasing nutritional security of the global population. Here, we refer “small-seeded grasses” including sorghum, pearl millet, finger millet, and other small millet crops by the term “millets.” We provide an update on the emerging trends, high-throughput phenotyping approaches, and the potential of genetics- and genomics-based strategies to address hidden hunger within a limited time frame using nutrient-rich millets as model crops.

## Relevance of millets in the human diet

**Figure 1:**
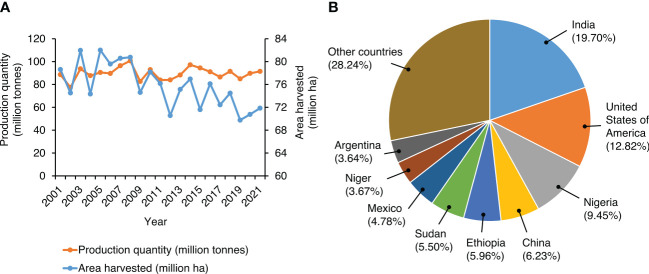
Global trends in millets production and cultivation. **(A)** Worldwide millet production in the last two decades. The graph represents production quantity (million tons) and the area under cultivation (million ha) from 2001 to 2021. Data represent the sum of values reported for millets and sorghum by FAOSTAT (2021). **(B)** Relative importance of the leading millet producing countries in the world. Data represent the sum of values reported for millets and sorghum production in 2021. *Source:* FAOSTAT (2021).

The major cereal crops and millets that are grown on hundreds of millions of hectares worldwide (www.fao.org/faostat; **Figure 1**) are vital to diets, cultures, and economies around the world, especially in populous developing regions. The global demand for all cereals and millets is rising and offers critical entry points for improving nutrition (Shiferaw et al., 2011; Shewry and Hey, 2015). They contain enhanced levels of proteins, vitamins, minerals, and antioxidants, which offer nutritional superiority over other grain crops. These crops also possess high levels of low glycemic index non-starchy polysaccharides and dietary fibers, apart from their enhanced micro- and macronutrient content. In this section, we describe the composition of key nutrients and minerals in millets and their relevance in human diets.

An extraneous group of forage grasses that produce small-sized grains are referred to as “millets.” Millets provide a rich source of nutrition, show minimum vulnerability to pathogens, and are tolerant to abiotic factors including drought and salinity (Muthamilarasan et al., 2016; Govindaraj et al., 2020; Vetriventhan et al., 2020; Babele et al., 2022). As a result, millets serve as ideal staple crops for the semi-arid tropics of Asia and Africa. They are highly nutritious and superior to rice and wheat because they are rich in proteins, fibers, minerals, and vitamins (Saleh et al., 2013). Approximately 80% of millets production is utilized for human consumption, whereas the remaining is used for livestock feed and beer production (Saleh et al., 2013; Shivran, 2016). Millet grains are highly endorsed for the well-being of children, adolescent girls, lactating mothers, elderly, and convalescents.

Sorghum is grown on over 40.93 million hectares of land globally, producing 61.36 million tons, while in India, it is cultivated on approximately 4.24 million hectares and produces 4.81 million tons (FAOSTAT, 2021). Sorghum is one of the cheapest sources of nutrition, provides a high amount of energy, protein, Fe, and Zn, and contributes to >50% of the Fe and Zn requirement (Ashok Kumar et al., 2011; Ashok Kumar et al., 2013). In general, sorghum grain contains 79%–83% starch, 7%–14% protein, and 1%–7% fat (Rhodes et al., 2017). Sorghum is mostly used for food purposes (55%), consumed in the form of flat breads and porridges (thick or thin), and the vegetative part of the sorghum plant is used as stover for livestock, especially in drylands. The nutritional value and chemical composition of sorghum are similar to major cereals including rice, wheat, and maize. The energy value (per 100 g) of sorghum grain lies between 296 and 356 kcal and constitutes mainly polysaccharides such as starch and non-starch, proteins, and lipids (**Table 1**). Mkandawire et al. (2013) demonstrated that sorghum contains the lowest starch digestibility value when compared to other carbohydrate-rich crops. For instance, most of the starch particles of sorghum are gradually digestible (30.0%–66.2%), whereas others are quickly digestible (15.3%–26.6%) or resilient (16.7%–43.2%). The higher proportion of slowly digestible and resistant starch in sorghum has an added advantage, as it offers a low glycemic index and less threat of chronic diseases like type II diabetes and obesity (Simnadis et al., 2016). Non-starch polysaccharides in sorghum consist of 75%–90% insoluble fibers (mainly arabinoxylans) and 10%–25% soluble fibers (Martino et al., 2012). The lipid content of sorghum is low (1.24–3.07 g/100g) and contains approximately 83%–88% unsaturated fatty acids. Furthermore, the levels of mono-unsaturated fatty acids in several sorghum varieties are lower than those of polyunsaturated fatty acids, and linoleic (45.6%–51.1%), oleic (32.2%–42.0%), and palmitic (12.4%–16.0%) acids are some of the major fatty acids detected in sorghum grains (Afify et al., 2012). As a result, the natural variation available for protein, fat, and starch content in sorghum can be exploited to improve nutritional quality through crop improvement programs.

**Table 1:** Nutritional status of millet grains compared with other cereal crops (per 100 g).

Nutrients	Sorghum	Millet	Rice	Wheat	Maize
Water	12.4 g	8.67 g	12.9 g	10.9 g	10.8 g
Energy	329 kcal	378 kcal	360 kcal	339 kcal	364 kcal
Protein	10.6 g	11 g	6.61 g	13.7 g	8.75 g
Total lipid (fat)	3.46 g	4.22 g	0.58 g	2.47 g	5.09 g
Ash	1.43 g	3.25 g	0.58 g	1.78 g	1.44 g
Carbohydrates	72.1 g	72.8 g	79.3 g	71.1 g	73.9 g
Dietary fiber	6.7 g	8.5 g	1.4 g	–	8.4 g
Sugars	2.53 g	–	–	–	–
Minerals					
Calcium, Ca	13 mg	8 mg	9 mg	34 mg	5 mg
Iron, Fe	3.36 mg	3.01 mg	4.36 mg	3.52 mg	1.74 mg
Magnesium, Mg	165 mg	114 mg	35 mg	144 mg	110 mg
Phosphorous, P	289 mg	285 mg	108 mg	508 mg	263 mg
Potassium, K	363 mg	195 mg	86 mg	431 mg	381 mg
Sodium, Na	2 mg	5 mg	1 mg	2 mg	5 mg
Zinc, Zn	1.67 mg	1.68 mg	1.16 mg	4.16 mg	2.24 mg
Copper, Cu	0.284 mg	0.75 mg	0.11 mg	0.553 mg	0.154 mg
Manganese, Mn	1.6 mg	1.63 mg	1.1 mg	3.01 mg	0.54 mg
Vitamins					
Thiamin (B1)	0.332 mg	0.421 mg	0.578 mg	0.419 mg	0.16 mg
Riboflavin (B2)	0.096 mg	0.29 mg	0.048 mg	0.121 mg	0.23 mg
Niacin (B3)	3.69 mg	4.72 mg	5.09 mg	6.74 mg	2.6 mg
Pantothenic acid (B5)	0.367 mg	0.848 mg	1.34 mg	0.935 mg	0.55 mg
Vitamin B6	0.443 mg	0.384 mg	0.145 mg	0.419 mg	0.47 mg
Folate (B9)	20 µg	85 µg	231 µg	43 µg	–
Vitamin B12	0 µg	0 µg	0 µg	0 µg	–
Vitamin C	0 mg	0 mg	0 mg	0 mg	–
Vitamin E	0.5 mg	0.05 mg	0.11 mg	–	–
Vitamin K	–	0.9 µg	–	–	–

Source: USDA FoodData Central Database (https://fdc.nal.usda.gov/fdc-app.html#/).

Other millets possess 10 genera and at least 14 species, of which pearl millet (*Pennisetum glaucum*) belongs to Paniceae tribe of the family Poaceae (Vetriventhan et al., 2020) and occupies 95% of the production (Nedumaran et al., 2014). Pearl millet contains high levels of micronutrients including Fe, Zn, and lysine (17–65 mg/g of protein) relative to other millet crops (McDonough et al., 2000; Haldimani et al., 2001). It is a good source of carbohydrates, resistant starch, proteins, dietary fibers, α-amylase activity, minerals, vitamins (A and B), and antioxidants, among others (Goswami et al., 2020) (**Table 1**). Increased levels of unsaturated fatty acids (75%) and phytic acid in pearl millet grains serve as a valuable resource for lowering cholesterol and phytate levels in individuals, which in turn decreases cancer risk. It contains high levels of antioxidants such as polyphenols, anthocyanins, tannins, phytates, and pinacosanols, which play a crucial role in regulating the aging process. Pearl millet is free of gluten and is suitable for consumption by people suffering from celiac disease who are usually allergic to the gluten from wheat and other cereals. Due to its nutritional superiority, pearl millet is beneficial for individuals suffering from diseases like diabetes, obesity, heart disorders, and atherosclerosis (Satyavathi et al., 2021). Furthermore, one of the key millet crops, finger millet (*Eleusine coracana* (L.) Gaertn.), serves as an important source of nutrition for people living in the developing world (Vetriventhan et al., 2016; Hittalmani et al., 2017). Finger millet is an important source of key nutrients including 18% dietary fiber, 6%–13% protein, 2.5%–3.5% minerals, 0.3%–3% phenolic compounds, and 0.34% calcium (Chandra et al., 2016). The crop is also valued for its health benefits like anti-diabetic, anti-tumorigenic, antioxidant, and antimicrobial properties (Nakarani et al., 2020; Kumar et al., 2021). Millets serve as a rich source of vitamins and trace elements that are essential for normal physiological functions in human. Among different millet crops, foxtail millet has maximum thiamine content (0.59 mg/100g), whereas proso millet is rich in riboflavin (0.28 mg/100g), which is very high compared to the riboflavin content of rice (0.04 mg/100g) and wheat (0.1 mg/100g). Furthermore, kodo millet contains high levels of iron (4.0 mg/100g), which is followed by finger millet (3.4 mg/100g), and foxtail millet (2.7 mg/100g) (Chandel et al., 2014). In addition, zinc levels were also found to be higher in foxtail millet (4 mg/100g), followed by barnyard millet (3.6 mg/100g) and finger millet (2 mg/100g). Taken together, these studies indicate that the micronutrient composition of millets are several fold higher as compared to the average micronutrient content in key non-millet cereals, thereby offering an inexpensive and sustainable solution to malnutrition.

## Precision phenotyping for grain micronutrient content

Precision phenotyping for grain micronutrient content is the key for the success of millet biofortification programs. Phenotyping for micronutrient traits such as Fe and Zn is challenging, as they are accumulated in minute quantities in the grains. Various phenotyping methods are being used to measure Fe and Zn concentrations, which include simple staining procedures to complex analytical protocols. The use of Perl’s Prussian blue for Fe and diphenyl thiocarbazone-based dithizone (DTZ) for Zn is a simple technique that gives a rough qualitative estimation of Fe and Zn in grain (Elango et al., 2021). Furthermore, elemental analysis techniques such as atomic absorption spectrometer (AAS), inductively coupled plasma–optical emission spectrometer (ICP-OES), micro-X-ray fluorescence spectroscopy (μ-XRF), secondary ion mass spectrometry (NanoSIMS) synchrotron X-ray, near-infrared reflectance spectrophotometer (NIRS), and fluorescence spectroscopy have been used to precisely measure grain micronutrient content (Rai et al., 2012; Shobana et al., 2013; Govindaraj et al., 2022). For instance, Govindaraj et al. (2016) employed X-ray fluorescence spectrometry (XRF) and ICP-OES methods for estimating Fe and Zn densities in diverse pearl millet genotypes and identified highly significant and positive correlations between these two advanced techniques for Fe (r = up to 0.97, *p*<0.01) and Zn (r = up to 0.98, *p*<0.01) contents. Furthermore, the application of high-resolution nanoscale secondary ion mass spectrometry (NanoSIMS) allows to qualitatively and quantitatively map the distribution of these micronutrients across the sorghum kernel components (Gaddameedi et al., 2022). Importantly, this knowledge will help reduce the micronutrient loss during seed and food processing.

In millets, the AAS, ICP-OES, and XRF methods are widely used for assessing grain Fe and Zn levels (Ashok Kumar et al., 2015; Pujar et al., 2020). In the case of finger millet, mineral and protein content were analytically determined using ICP-OES (Puranik et al., 2020). In addition, seed nitrogen content was determined using combustion, which was then followed by thermal conductivity using the Leco FP-528 Nitrogen/Protein Determinator (Puranik et al., 2020). Among all, XRF is a low-cost and high-throughput method for assessing grain Fe and Zn, and there is good correspondence between ICP-OES and XRF methods for assessing the grain Fe and Zn, but ICP-OES is more accurate. Hence, one can use XRF to discard the lines, segregate populations with low Fe and Zn, and validate all high Fe and Zn lines with ICP-OES method. Recently, Vetriventhan et al. (2021) reported the use of ICP-OES method to estimate Fe, Zn, and Ca content in 200 diverse little millet landraces. Of the total number of accessions evaluated, approximately 80% of accessions revealed consistent protein and Zn content, whereas less consistency was observed for Fe (64%) and Ca (30%) content. Notably, significant positive correlation (*R* ^2 =^ 0.69–0.74, *p ≤* 0.001) was observed for trait-specific accessions possessing higher grain weight (10 accessions), grain yield (15), biomass (15), and having consistently higher grain nutrient levels (30) over a 2-year period. In addition, five promising accessions possessing higher grain yield and Ca content were also identified (Vetriventhan et al., 2021). After multi-location field evaluation, promising accessions with superior agronomic performance and higher grain nutrient levels can be released for commercial cultivation or used in millet improvement programs.

## Genetic resources for nutritional traits discovery and utilization

Genetic variability acts as a raw material, and its utilization by plant breeders is a key step in biofortification programs. Genetically diverse accessions for micronutrient availability that are conserved in genebanks serve as a rich source of genetic resources for designing nutrient-dense and high-yielding crops for food and nutritional security. Understanding the extent of genetic variability for micronutrients in plant genetic resources along with underlying genetics of accumulation mechanisms is critical for the development of nutrient-rich varieties.

Sorghum is a highly diverse species with approximately 256,000 germplasm accessions conserved globally, and the genebank at ICRISAT conserves the largest collection of over 42,000 accessions originating from 92 countries. These accessions are well characterized for various morpho-physiological and agronomic traits (Ashok Kumar et al., 2013). The core collection (2,247 accessions) and mini core collection (242 accessions) representing the global sorghum collection conserved at the ICRISAT genebank have been developed (Grenier et al., 2001; Upadhyaya et al., 2009). On accessing the Fe and Zn concentrations of all the accessions belonging to the core collection, large variability was found for Fe and Zn content ranging from 26–60 mg kg^−1^ and 21–57 mg kg^−1^, respectively (Ashok Kumar et al., 2012). In addition, when compared to the germplasm accessions, cultivars and breeding lines were found to possess less Fe and Zn levels (Reddy et al., 2005). The quantitative inheritance nature of these traits could be the possible reason for the observed variability in Fe and Zn concentration. On the other hand, the lower concentration of these two minerals in cultivars and breeding lines relative to landraces can be explained by the lack of breeding interest in these traits until the end of the second millennium. Based on the consumption level of sorghum (200 g) and estimated bioavailability of Fe (10%) and Zn (25%) in sorghum, it is necessary to enhance the Fe concentration by 30 mg kg^−1^ and zinc concentration by12 mg kg^−1^ over the base levels (30 mg kg^−1^ of Fe and 20 mg kg^−1^ of Zn) to meet the RDA requirements. In addition to the variability observed in grain Fe and Zn content, a huge variability was detected in grain phytate content, but not much variability in β-carotene content. On studying diverse sorghum cultivars (yellow endosperm lines, germplasm lines, high-protein digestible lines, high-lysine lines, and waxy lines), significant genetic differences were observed for Fe, Zn, and phytate concentrations and agronomic and grain quality traits (Reddy et al., 2005). Large genetic variability for grain Fe and Zn concentrations was also noted in sorghum hybrid parents (>500 B-lines and 100 R-lines) and 67 commercial hybrids (Ashok Kumar et al., 2009; Ashok Kumar et al., 2012). Ng'uni et al. (2012) reported that grain Fe concentration ranged from 2.8 to 6.3 mg/100 g, and grain Zn content varied from 2.3 to 5.5 mg/100 g in South African sorghum cultivars. A large genotype × environment interaction was observed for Fe and Zn content in sorghum. That said, top-ranking genotypes excelled in most environments and years (Ashok Kumar et al., 2013). Hence, multi-location and multi-season evaluation is critical for the effective phenotyping for grain Fe and Zn concentrations to identify stable lines.

Many pearl millet germplasm collections are available worldwide, which includes the Pearl Millet inbred Germplasm Association Panel (PMiGAP) (Varshney et al., 2017). The PMiGAP has been developed at ICRISAT, India based on a core collection of 1,000 accessions, including landraces and cultivars originating from across three continents and representing genetic diversity within 27 countries (Sehgal et al., 2015; Srivastava et al., 2020). The PMiGAP has been completely re-sequenced with approximately 29 million genome-wide single nucleotide polymorphism (SNPs) (Srivastava et al., 2020). Besides this, the Iniari germplasm collection developed by ICRISAT includes landraces originating from West Africa, which possess high grain filling capability under terminal water deficit, big seeds, long panicles, and wide leaves (Ito et al., 1999). Taking advantage of cross-pollination driven by the protogynous flowers in pearl millet, breeders are in a continuous search for identifying nutritionally elite varieties suited for local environments. Recent studies have demonstrated high variability for grain Fe and Zn content in different breeding materials (Rai et al., 2014a; Rai et al., 2016; Upadhyaya et al., 2016). For instance, Rai et al. (2014a) screened seeds of parental progenies and restorer parent progenies for evaluating Fe and Zn variability using X-ray fluorescence spectroscopy. The mean Fe density of these progenies was found to be 5%–66% higher compared to the control cultivars. Furthermore, Rai et al. (2016) and Upadhyaya et al. (2016) used inductively coupled plasma atomic emission spectroscopy to scrutinize diverse germplasm accessions and landraces and observed substantial variability for Fe (51–121 mg kg^−1^) and Zn (46–87 mg kg^−1^). In finger millet, grain nutrients assessment of the core germplasm collection (622 accessions) revealed substantial variability for Fe (21.71–65.23 mg kg^−1^), Zn (16.58–25.33 mg kg^−1^), Ca (1.84–4.89 g kg^−1^), and protein (6.00–11.09%) (Upadhyaya et al., 2011a). In another study, Puranik et al. (2020) also reported substantial variability in finger millet (190 accessions) for calcium (223.63–422.56 mg 100 g^−1^), potassium (266.60–668.83 mg 100 g^−1^), magnesium (106.36–179.99 mg 100 g^−1^), and protein content (3.86–11.27% w/w). Similarly, grain nutrients assessment of a diverse set of germplasm including core collection conserved at the ICRISAT genebank revealed a significant variability for grain nutrients in foxtail millet [Fe (24.1–68.0 mg kg^−1^), Zn (33.6–74.2 mg kg^−1^), Ca (90–288 mg kg^−1^), and protein (10.7–18.5%)] (Upadhyaya et al., 2011b), proso millet [Fe (41–73 mg kg^−1^), Zn (26–47 mg kg^−1^), Ca (91–241 mg kg^−1^), and protein (11–19%)] (Vetriventhan and Upadhyaya, 2018), kodo millet [Fe (14.4–56.4 mg kg^−1^), Zn (17.0–31.5 mg kg^−1^), Ca (121–321 mg kg^-1^), and protein (5.6%–11.3%)] (Vetriventhan and Upadhyaya, 2019), and little millet [Fe (17.6–58.0 mg kg^−1^), Zn (19.4–39.5 mg kg^−1^), Ca (92–390 mg kg^−1^), and protein (6–15.6%)] (Vetriventhan et al., 2021). In the case of foxtail millet, a large variability was detected in the diversity panel (93 accessions) for potassium (1,477.9–3,365.5 ppm), magnesium (764.7–1,795.3 ppm), phosphorus (1,756.0–4,135.0 ppm), and sulfur content (703.0–1,908.8 ppm) (Jaiswal et al., 2019). Taken together, these studies reveal that continuation of breeding program with planned crosses will pave the way towards detecting nutrient-rich parental genotypes suitable for fluctuating environmental conditions.

## Breeding approaches for enhancing micronutrient content in millets

### Conventional breeding

The conventional breeding approach involves the selection and crossing of two parental genotypes possessing high micronutrient concentrations (e.g., Fe and Zn) to develop a hybrid expressing the desired trait(s). Natural genetic variation for target traits existing within the crop gene pool holds the key to successful crop genetic improvement through conventional breeding approaches.

Biofortified (Zn and Fe rich) sorghum lines (e.g., ICSR 14001 also known as “*Parbhani Shakti”*) and hybrids (ICSH 14002, ICSA 661 × ICSR 196, ICSA 318 × ICSR 94, and ICSA 336 × IS 3760) have been jointly developed by ICRISAT-Vasantrao Naik Marathwada Krishi Vidyapeeeth (VNMKV) and released for commercial cultivation in India. Furthermore, two sorghum varieties, improved Deko (12KNICSV-188) and improved Zabuwa (12KNICSV-22), possessing high Fe and Zn content have been released in Nigeria (Okwuonu et al., 2021). The improved Deko variety has Fe content (126 ppm), which is three times higher than that obtained from local sorghum varieties (40 ppm) (ICRISAT, 2016). These improved varieties have the potential to boost the nutritional levels of malnourished populations, especially children, in Nigeria. These new varieties involved the crossing of local Nigerian germplasm with improved lines from ICRISAT (Mali). Similarly, in India, a biofortified (Fe and Zn rich) pearl millet variety (named *Dhanashakti*) and a hybrid ICMH 1201 (named Shakti-1201) were released by ICRISAT and HarvestPlus in 2014. In addition, two varieties, namely, ICMH 1202 (Nirmal-7) and ICMH 1301, are being evaluated under advanced varietal trials. Several well-adapted commercial pearl millet varieties and their progenies and hybrids with high grain Fe and Zn content have also been previously reported (Velu et al., 2007; Rai et al., 2012). However, breeding for higher levels of grain micronutrient content also depends on the existing environmental conditions, including soil mineral composition, which further complicates the process (Feil et al., 2005). As a result, the stability of phenotyping data should be verified through multi-environmental trials while breeding biofortified crops for future climates.

### Integrated *omics* approaches

**Figure 2:**
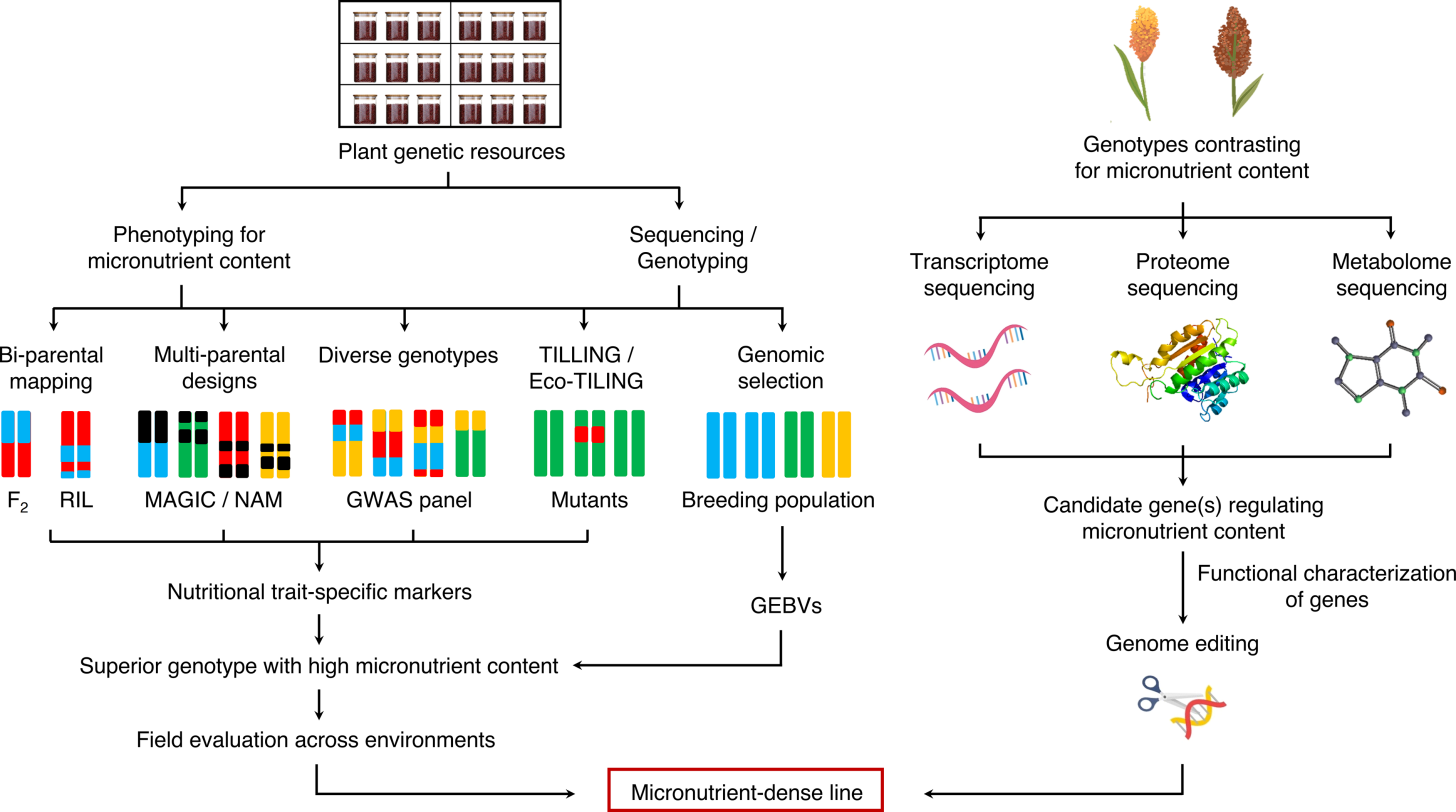
Genomic breeding strategies for enhancing nutritional content in millets. RIL, recombinant inbred line; MAGIC, multi-parent advanced generation inter-cross; NAM, nested association mapping; GWAS, genome-wide association study; TILLING, targeting induced local lesions in genomes; GEBV, genomic estimated breeding value.

The conventional breeding approaches have been successful in the past decades in developing biofortified millet varieties (Rai et al., 2014b; Satyavathi et al., 2021). However, these approaches alone are not sufficient to keep pace with the future food and nutritional demands of the burgeoning population. The realization of biofortified sorghum and millet varieties for future climates can be significantly accelerated if assisted by recent innovations in omics approaches (Varshney et al., 2021a). Furthermore, a huge genetic variation for micronutrient concentration prevails among the sorghum and millet germplasm; however, it is yet to be characterized in detail. Evaluating the available germplasm accessions for micronutrient concentrations and detection of high-fidelity molecular markers through genome-wide association studies (GWAS) will facilitate the identification of quantitative trait loci (QTLs)/candidate genes/alleles regulating nutritional traits of interest. The identified QTLs/genes can then be introgressed into elite cultivars *via* genomics-assisted breeding or transgene-based methods to enhance the grain micronutrient content (Varshney et al., 2021b). The genetic relatedness of millets with other cereals, such as rice, wheat, and barley, further enables introgression of these nutritional traits into major cereal crops. The integration of different omics approaches such as genomics, transcriptomics, proteomics, metabolomics, and ionomics is crucial in designing biofortified crops for the future (**Figure 2**).

#### Genomics: QTL mapping and genome-wide association studies

Radical developments in genomics techniques have led to the development of various marker systems including mapped microsatellite or simple sequence repeat (SSR) markers, SNPs markers, insertion–deletion (InDel) markers, and more recently haplotype-based SNP markers. Linkage mapping and association mapping are the two most used tools for dissecting the genetics of complex nutritional traits in plants (Roorkiwal et al., 2022). Traditional linkage mapping/QTL mapping explores the recombination events and marker–trait associations (MTAs) in bi-parental segregating populations, such as F_2_, doubled haploid (DH), and recombinant inbred lines (RILs). This method is very powerful in capturing major genes with larger effects and rare alleles (Kumar et al., 2016). On the other hand, GWAS explores functional variations within genetically diverse panels through linkage disequilibrium (LD) analysis, which is very efficient and effective for identifying new genomic regions (Yu and Buckler, 2006; Roorkiwal et al., 2022). Here, we highlight some key genomics efforts undertaken in millets to identify genomic regions associated with grain nutrient content (**Table 2**).

**Table 2:** A list of some key QTLs identified for grain nutrient content in millets.

Crop	Trait	Mapping population	Population type	QTL(s)/MTAs/nearest marker	PVE (%)	Reference
Sorghum	Fe content	296B × PVK 801	RIL	*qfe1.1, qfe6.1, qfe7.1, qfe7.2*	5.09-6.80	Kotla et al., 2019
	Zn content	296B × PVK 801	RIL	*qzn7.1, qzn7.2, qzn7.3, qzn7.4*	5.70-9.42	Kotla et al., 2019
	Starch content	Rio × BTx623	RIL	7 QTLs	6.00-26.00	Murray et al., 2008
	Fat content	Rio × BTx623	RIL	3 QTLs	8.00-14.00	Murray et al., 2008
	Crude protein content	Rio × BTx623	RIL	7 QTLs	8.00-24.00	Murray et al., 2008
	Lutein content	KS115 × Macia	RIL	Lu-1.1, Lu-1.2, Lu-1.3, Lu-2.1, Lu-3.1, Lu-10b.1	7.13-28.50	Salas Fernandez et al., 2008
	Zeaxanthin content	KS115 × Macia	RIL	Ze-6.1, Ze-6.2	18.18-25.40	Salas Fernandez et al., 2008
	β-carotene content	KS115 × Macia	RIL	Bc-1.1, Bc-2.1, Bc-2.2, Bc-2.3, Bc-10b.1	8.00-15.15	Salas Fernandez et al., 2008
	Total carotenoids	KS115 × Macia	RIL	Tc-2.1, Tc-2.2	7.72-20.12	Salas Fernandez et al., 2008
	β-carotene content	–	Association mapping panel	14 MTAs	–	Cruet-Burgos et al., 2020
	Zeaxanthin content	–	Association mapping panel	38 MTAs	–	Cruet-Burgos et al., 2020
	Protein content	–	Association mapping panel	4 MTAs	34.00-36.00	Rhodes et al., 2017
	Fat content	–	Association mapping panel	41 MTAs	29.00-35.00	Rhodes et al., 2017
	Fe content	–	Association mapping panel	Sobic.001G213400	–	Shakoor et al., 2016
	Zn content	–	Association mapping panel	Sobic.007G064900	–	Shakoor et al., 2016
	Mn content	–	Association mapping panel	Sobic.003G349200	–	Shakoor et al., 2016
	Mg content	–	Association mapping panel	Sobic.001G443900	–	Shakoor et al., 2016
	Cd content	–	Association mapping panel	Sobic.002G083000	–	Shakoor et al., 2016
Pearl millet	Fe content	PPMI 683 × PPMI 627	RIL	14 QTLs	2.85-19.66	Singhal et al., 2021
	Zn content	PPMI 683 × PPMI 627	RIL	8 QTLs	2.93-25.95	Singhal et al., 2021
	Fe content	ICMB 841-P3 × 863B-P2	RIL	Xpsmp2214-Xipes142, Xpsmp322-Xipes181, pgpb11029-pgpb8456	18.10-19.40	Kumar et al., 2016
	Zn content	ICMB 841-P3 × 863B-P2	RIL	Xpsmp2214-Xipes142, Xpsmp2214-Xipes142, Xpsmp2040-pgpb10727	19.70-50.10	Kumar et al., 2016
	Fe content	ICMS 8511-S1-17-2-1-1-B-P03 × AIMP 92901-S1-183-2-2-B-08	RIL	11 QTLs	9.00-31.90	Kumar et al., 2018
	Zn content	ICMS 8511-S1-17-2-1-1-B-P03 × AIMP 92901-S1-183-2-2-B-08	RIL	8 QTLs	9.40-30.40	Kumar et al., 2018
	Fe content	–	Advanced inbred lines	18 MTAs	5.07-8.23	Pujar et al., 2020
	Zn content	–	Advanced inbred lines	43 MTAs	5.10-8.00	Pujar et al., 2020
	Protein content	–	Advanced inbred lines	17 MTAs	5.11-5.86	Pujar et al., 2020
	Fe content	–	Association mapping panel	21 MTAs	7.00-18.10	Anuradha et al., 2017
	Zn content	–	Association mapping panel	24 MTAs	8.20-16.50	Anuradha et al., 2017
Finger millet	Ca content	–	Association mapping panel	44 MTAs	4.80-17.79	Sharma et al., 2022
	Fe content^a^	–	Association mapping panel	895 MTAs	5.66-32.31	Puranik et al., 2020
	K content^a^	–	Association mapping panel	454 MTAs	5.78-19.89	Puranik et al., 2020
	Mg content^a^	–	Association mapping panel	5 MTAs	12.39-18.84	Puranik et al., 2020
	Na content^a^	–	Association mapping panel	643 MTAs	5.73-26.46	Puranik et al., 2020
	Zn content^a^	–	Association mapping panel	96 MTAs	7.55-17.07	Puranik et al., 2020
	Ca content	–	Association mapping panel	9 MTAs	7.90-41.00	Kumar et al., 2015
Foxtail millet	Fe content	–	Association mapping panel	1 MTA	–	Jaiswal et al., 2019
	Zn content	–	Association mapping panel	5 MTAs	–	Jaiswal et al., 2019
	Mg content	–	Association mapping panel	3 MTAs	–	Jaiswal et al., 2019
	B content	–	Association mapping panel	1 MTA	–	Jaiswal et al., 2019

a For finger millet, the number of MTAs indicate significant associations [−log_10_(p) ≥ 3.00; p ≤.001 (for GLM) and −log_10_(p) ≥ 2.00; p ≤.01 (for MLM), and an FDR < 0.1] reported by Puranik et al. (2020).

PVE, phenotypic variance explained; QTL, quantitative trait loci; MTA, marker trait association; Fe, iron; Zn, zinc; Mn, manganese; Mg, magnesium; Cd, cadmium; K, potassium; Na, sodium; Ca, calcium; B, boron.

One of the major concerns in sorghum is the limited bioavailability of micronutrients in the grain due to complexity of polyphenols and tannins. For the identification of QTLs for grain Fe and Zn content, Phuke et al. (2017) developed a RIL population from the cross 296B × PVK 801, with parents contrasting for grain Fe and Zn content. This study showed a wide variation in grain Zn (10.2–58.7 mg kg^−1^) and Fe (10.8–76.4 mg kg^−1^) content existing within the RIL population. The grain Fe and Zn content was found to be negatively correlated with yield and positively correlated with 100-seed weight, suggesting a selection pressure of bolder seeds in biofortification programs. A significant G×E interaction was observed for both the micronutrients, with Fe being highly influenced by the environment compared to Zn (Phuke et al., 2017). Furthermore, a genetic map was constructed for 296B × PVK 801 RIL population with 2,088 markers (1148 DArTs, 927 DArTSeqs, and 13 SSRs), covering a distance of 1,355.52 cM with an average marker interval of 0.6 cM (Kotla et al., 2019). Here, 11 QTLs (individual environments) and 3 QTLs (across environments) for Fe and Zn were identified. A common genomic region was identified for ICRISAT environment, which explained a phenotypic variance of 9.42% and 5.82% for Zn and Fe, respectively. After validation, the linked markers identified in this study can be deployed for developing high-grain Fe and Zn cultivars in sorghum improvement programs globally (Kotla et al., 2019). To unravel the genetic determinants of natural variation observed for seed element concentration, Shakoor et al. (2016) performed a GWA mapping of alleles regulating 20 traits influencing sorghum seed ionome, i.e., the mineral nutrient and trace element composition representing the inorganic component of cellular and organismal systems (Salt et al., 2008). This study also identified putative genes regulating Zn, Mn, Ni, Ca, and Cd accumulation in sorghum seeds.

Genetic variation observed among adapted pearl millet inbreds and hybrids suggests the possibility of improving grain micronutrient concentrations by selective breeding. In a previous study, Kumar et al. (2016) performed QTL mapping with 305 (96 SSRs; 208 DArT) markers distributed across seven linkage groups, covering a distance of 1,749 cM. Based on the phenotypic data collected across two different environments, QTLs for Fe and Zn content were found to co-localize on linkage group (LG) 3. QTL analysis using a pearl millet RIL population derived from PPMI 683 × PPMI 627 cross resulted in the identification of 14 QTLs for Fe and 8 QTLs for Zn with phenotypic variation ranging up to 19.66% and 25.95%, respectively (Singhal et al., 2021). These QTLs encompassed genes encoding ferritin, and Al^3+^, K^+^, Zn^2+^, and Mg^2+^ transporters. Furthermore, Anuradha et al. (2017) performed GWAS using an association mapping panel comprising of 130 diverse lines of pearl millet, showing a wide range of grain micronutrient content. This study identified a total of 16 genomic regions for grain Fe and Zn content. Some regions were consistent across locations and years, while others were specific to a particular location or year. Furthermore, a GWAS performed using 3 million SNPs generated using GBS resulted in the identification of several hundred significant MTAs for grain Fe and Zn content. This study also revealed six candidate genes linked with Fe/Zn uptake. The most significant candidate was found to be the YUCCA-11 gene, which is known to drive Zn efficiency by auxin biosynthesis (Manwaring, 2018). Furthermore, Pujar et al. (2020) performed GWAS analysis using a diverse panel of 281 advanced inbred lines to identify MTAs for grain Fe, Zn, and protein content. This study revealed 78 MTAs (including 18 MTAs for Fe, 43 MTAs for Zn, and 17 MTAs for protein content), and some promising candidate genes associated with grain Fe, Zn, and protein content in pearl millet. For finger millet, Puranik et al. (2020) identified 418 common MTAs associated with diverse mineral content using general linear model and mixed model approaches. Among these, 34 MTAs were above the Bonferroni threshold. From these 34 MTAs, 18 revealed homology with candidate genes involved in binding, remobilization, or metal ion transport (Puranik et al., 2020). After functional validation of these MTAs, these markers can be deployed in breeding efforts using genomic breeding approaches to develop high-grain nutritional quality in finger millet.

#### Transcriptomics

Recent advances in next-generation sequencing technologies have greatly revolutionized transcriptome sequencing. Being cost effective and with high coverage, transcriptomics has been performed in many crop species to identify candidate genes associated with nutrient biosynthesis and accumulation (Mishra et al., 2019). Additionally, RNA-sequencing (RNA-seq) provides information about the relative abundance of transcripts (at a given stage/condition) and enables molecular marker development in a high-throughput fashion. For instance, RNA-seq in grains of three sorghum cultivars differing in grain color led to the identification of >3,000 differentially expressed genes, which were mainly enriched in carbohydrates, amino acid, and flavonoid metabolism that may influence the grain nutritional content (Zhou et al., 2020). Furthermore, comparing the expression patterns of the genes (from an RNA-seq dataset) underlying a GWAS QTL led to the identification of a putative alpha-amylase 3 gene as a strong candidate associated with the variation in protein and fat content in sorghum grains (Rhodes et al., 2017). In a study done by Mahendrakar et al. (2020), in a set of contrasting mapping population parents for grain Fe and Zn content, diverse growth stages revealed tissue- and stage-specific expression patterns for a total of 29 Fe and Zn metabolism genes. Gene families including *PglZIP*, *PglNRAMP*, and *PglFER* were found to be candidates for grain Fe and Zn content, with ferritin-like gene, *PglFER1*, as the potential candidate gene for grain Fe content.

In a recent study, transcriptome profiling of stage-specific spikes of pearl millet genotypes contrasting for grain Fe and Zn content was performed to identify candidate genes expressed in developing spikes and those associated with the variation in Fe and Zn levels (Satyavathi et al., 2022). Here, 155 and 251 transcripts were found to be up- and downregulated, respectively, in the genotypes showing high Fe and Zn content, whereas 349 and 378 transcripts were differentially expressed during the flowering and milking stages of development, respectively. Gene Ontology analysis revealed that the genes involved in metabolic activities were primarily associated with uptake and transport of Zn and Fe in pearl millet (Satyavathi et al., 2022). Since finger millet grains contain higher levels of calcium content, RNA-seq of spike tissues of two finger millet genotypes contrasting for calcium content (GP-45, high calcium content; GP-1, low calcium content) was performed (Singh et al., 2014). A comparison of the relative abundance of transcripts revealed high expression of 24 calcium sensor genes (e.g., CaM, CaMLs, CBLs, CIPKs, and CRKs) in high calcium genotype. Collectively, these studies demonstrate the application of transcriptomics and encourage RNA-seq to be carried out in millets to comprehensively identify and functionally characterize the function of genes regulating nutritional content in these crops.

#### Proteomics and metabolomics

Proteomics and metabolomics represent some of the major players among omics approaches, as they are critical for characterizing the biomolecules and trace elements that have a high nutritional value. These approaches hold potential in identifying the protein function and in enhancing the production of crucial metabolic compounds in millets by offering key insights into the biological pathways. For example, in a recent study, non-targeted metabolomics analysis using a set of 61 diverse sorghum accessions enabled the differentiation of temperate and tropical sorghums based on the accumulation of phenolic acids, phytosterols, flavonoids, carotenoids, amino acids, sugars, and fatty acids (Ramalingam et al., 2021). This study offered new opportunities for generating biofortified sorghum varieties with enhanced nutraceutical and therapeutic characteristics. Although no major attempts have been made to analyze the nutrients at a protein and metabolite scale, these studies will pave the way for the identification of the proteins and metabolites in sorghum and/or millet seeds in response to several factors. Importantly, these approaches will facilitate the development of biomarkers for key nutritional traits in these crops. Taking into account the intricate and interrelatedness of physiological and metabolic pathways in crops, proteomics and metabolomics strategies will enable detection of putative genes having nutritive properties *via* a bottom–up approach. While the grains of millets are the major source of nutrition for people living in the developing countries, proteome and metabolome at diverse stages of seed development should be analyzed to detect candidate genes and their role in nutrient biosynthesis and accumulation.

#### Ionomics

With continuous technological innovations in genotyping and sequencing platforms, high-throughput phenotyping assumes higher importance for enhancing grain micronutrient content in different crops. To this end, ionomics has emerged as a high-throughput “elemental profiling” approach for accurately measuring mineral nutritional content of a living organism (Baxter, 2009; Huang and Salt, 2016). Precise and accurate estimation of grain micronutrients is crucial for expediting the identification of genotypes possessing high micronutrient content (Swamy et al., 2016). A large number of elemental analysis techniques such as AAS, ICP-OES, µ-XRF, and NIRS have been used to correctly measure grain micronutrient levels (Trijatmiko et al., 2016; Manickavelu et al., 2017; Khokhar et al., 2018). In this context, community-oriented databases such as ionomic HUB or iHUB (http://www.ionomicshub.org/home/PiiMS) have been established to allow researchers to freely access ionomic resources of different plants including *Arabidopsis*, rice, and soybean (see Baxter et al., 2007). From the nutrition point of view, ionomics will serve as an effective approach to identify mineral transport mechanisms in millets by detecting the transporter genes and characterizing their molecular functions (Kumar et al., 2014). Although ionomic studies in crops are still in their preliminary stage, the role of ionome can be extrapolated to identify nutrient levels in grain crops. This information will in turn facilitate the development of biofortified crops and the required experimental work plan to enable efficient bioavailability of nutrients.

### Transgene-based approaches

Transgenic approaches can be utilized to simultaneously integrate genes involved in improving micronutrient concentration and their bioavailability and reducing anti-nutrient concentration that limit the bioavailability of nutrients in plants. In addition, genetic engineering can also be used to redistribute micronutrients among tissues, enhance micronutrient levels in edible parts, and modulate biochemical pathways to increase grain micronutrient concentration in commercial crops (Sharma and Agarwal, 2005). Although the development of biofortified crops using genetic engineering is a time-consuming, labor-intensive, and costly effort during the research and development stage, eventually, it serves as a cost-effective and sustainable strategy, unlike conventional biofortification programs (White and Broadley, 2005). Transgenic crops with enhanced micronutrient contents hold the key to address hidden hunger especially among the smallholder farming households and vulnerable populations (women and young children) in the developing countries (Hirschi, 2009).

To date, there is no major study available for improvement of Fe and Zn by transgenic methods in millets. That said, sorghum has been targeted to improve provitamin A (beta-carotene) concentration by expressing Homo188-A (Lipkie et al., 2013). A biofortified sorghum with enhanced and stabilized pro-vitamin A providing 20%–90% of the estimated average requirement (EAR) for children under age 3 was developed recently using genetic transformation (Zhao et al., 2019). Moreover, it was demonstrated that provitamin A can be stabilized in sorghum by the co-expression of vitamin E through ectopic expression of homogentisate geranylgeranyltransferase (HGGT) and that vitamin E can enhance the stability of provitamin A *in planta*. These findings have the potential to impact directly the lives of millions of people who suffer from vitamin A deficiency, and they can be applied to enhance provitamin A stability in many food crops (Che et al., 2016). Similarly, the content of essential amino acid lysine was improved in sorghum by the introduction of a genetically engineered high lysine protein in the living sorghum cells (Zhao et al., 2003).

#### Genome editing

A recent development in the genome editing toolbox, Clustered Regulatory Interspaced Short Palindromic Repeats (CRISPR)/CRISPR-associated nuclease protein (Cas) systems for precise modification within the genome, gives researchers a possibility for accurately targeting the genes or genomic regions of interest (Zhu et al., 2020). CRISPR/Cas-mediated genome editing has been successfully used in major cereal crops; however, most millets have largely remained eluded from this success (Pixley et al., 2022). This is mainly because of the availability of limited genomic resources and lack of precise transformation systems developed in millets. A completely annotated whole-genome sequence is a major prerequisite for predicting the target genomic regions and designing guide RNA for the CRISPR/Cas system. Among millet crops, since a complete and annotated genome sequence is available only for foxtail millet to date (Vetriventhan et al., 2020), the off-target effects of genome-edited plants could not be studied precisely in other millets. Therefore, an effective application of genome editing systems in the future will rely mainly on the complete and annotated genomes of millets.

Notably, genome editing can aid in unravelling the mechanisms underlying nutrient fortification and transferring these key traits to major cereals. The development of nutrient-rich millet grains can also benefit from this method by tweaking the expression of genes involved in homeostasis and/or by editing the regulatory element of homeostasis genes. For instance, CRISPR/Cas9 approach is being used to target the k1C genes to create variants with reduced kafirin levels and improved protein quality and digestibility and improved lysine content in sorghum (Li et al., 2018). In addition, remarkably higher levels of mineral nutrients such as Ca (finger millet), Fe (barnyard millet), and vitamin B in millet seeds can be explored using genome editing tools to discover the transporters and signaling genes controlling seed biofortification. These traits can be transferred from millets to mainstream cereals to strengthen food sand nutritional security. In millets, biofortification is mainly limited due to the presence of antinutritional factors such as phytic acid, tannins, and polyphenols present in grains (Boncompagni et al., 2018). To this end, precision genome editing approaches like base editing can be used to reduce the quantity of antinutrients and enhance the bioavailability of macro- and micronutrients in millet grains. Taken together, the availability of high-quality reference genomes and efficient transformation systems could galvanize biofortification in millets using advanced genome editing tools, for sustainable food and nutritional security in the dryland tropics of South Asia and sub-Saharan Africa.

## Conclusion and future perspectives

Millets have the nutritional potential for feeding vulnerable and poor populations in the semi-arid tropics of South Asia and sub-Saharan Africa. However, research efforts targeted towards exploring and utilizing nutrient-dense millet crops to address micronutrient malnutrition are still inadequate. Although millet grains possess significantly higher levels of essential amino acids, vitamins, and minerals, the bioavailability of nutrients in grains requires further research for improvement. It is now well known that biofortification serves as a promising and cost-effective approach to increase micronutrient content in food crops for enhancing the nutritional status of target populations (especially young children, adolescent girls, and women) across the world. Globally, committed efforts by HarvestPlus, ICRISAT, CGIAR, and other international and national initiatives are serving as pillars to achieve these goals. However, biofortification in millets is a challenging endeavor to make an impact in non-traditional areas and urban markets. Precision phenotyping for grain micronutrient content and characterizing diverse millet germplasm at the genotypic and phenotypic levels (for nutritional traits in combination with adaptive traits) would be useful for discovering novel sources of variation in nutritional traits. Recent innovations in next-generation sequencing technologies will enable the development of high-quality reference genomes for millets in a faster and more precise way. As a result, the identification of closely linked markers for nutritional traits will accelerate mainstreaming efforts to develop biofortified varieties and will also uncover the candidate genes controlling these traits. Integration of different omics approaches/genome editing with conventional biofortification programs to a larger extent can reflect in the rapid delivery of nutrient-dense millet varieties to address future food and nutritional security. Furthermore, since millets display cross-genera transferability, introgression of genomic regions/candidate genes associated with key nutritional traits from millets into mainstream cereals will be facilitated using genomics-assisted breeding or genome editing approaches. Considering their potential in addressing micronutrient malnutrition and hidden hunger among poor populations from developing countries, biofortified millets developed through genetic and genomic interventions hold a bright future.

## Author contributions

HK and MG conceptualized the idea. HK, RB, HV, SG, and RP wrote the manuscript. HK, RB, VM, RS, PJ, EH, SG, SKG, and MG revised and finalized the manuscript. All authors contributed to the article and approved the submitted version.
